# Valvular Excrescences: A Possible Transient Phenomenon

**DOI:** 10.1155/2015/380765

**Published:** 2015-11-16

**Authors:** Peter Marstrand, Maiken Brit Jensen, Nikolaj Ihlemann

**Affiliations:** ^1^Department of Cardiology, The Heart Centre, University Hospital of Copenhagen, Blegdamsvej 9, 2100 Copenhagen, Denmark; ^2^Department of Thoracic Anaesthesiology, The Heart Centre, University Hospital of Copenhagen, Blegdamsvej 9, 2100 Copenhagen, Denmark

## Abstract

The thrombogenic potential of Lambl excrescences (LE) is minimal unlike the benign tumour fibroelastoma wherefrom thrombi often originate. We present a patient with multiple strokes within a six-year period. A possible locus on the aortic valve was found and diagnosed as fibroelastoma. Before aortic valve substitution prior finding could not be visualized and surgery was aborted. Due to review of earlier Transesophageal Echocardiography (TEE) and the transient component, LE was accepted as the most plausible diagnosis. This illustrates the need for TEE just before surgery.

## 1. Introduction

A vegetation/excrescence on the valves of the heart is a relatively normal finding when performing echocardiography and is visualized in up to 40% of healthy individuals [1]. The vegetation/excrescence are mainly caused by Lambl excrescences (LE). In 1856 the Czech pathologist Vilém Dušan Lambl first described the presence of filiform fronds localized on the closure line of the ventricle side of the aortic valve [2]. In the literature LE are often referred to as “valvular strands” and the matrix consists of mucopolysaccharide covered with layers of fibrin and a single layer of endothelium towards the lumen [3]. LE are described with various prevalences and are mostly accepted as a benign phenomenon with a transient potential. However, there are case reports where a connection between LE and thromboembolic events has been suggested. As differential diagnosis infectious vegetations, endocarditis and fibroelastoma should be considered. Fibroelastoma, the most frequent valvular tumour, is broadly based and described as “sea anemone-like” due to the gelatinous papillary mass [4]. Unlike LE, fibroelastoma is thrombogenic if sized >1 cm and if embolic events occur; surgical removal is recommended [5]. Infectious vegetations must always be considered if the patient presents a history with fever and positive blood cultures.

## 2. Case Report

**Figure 1:**
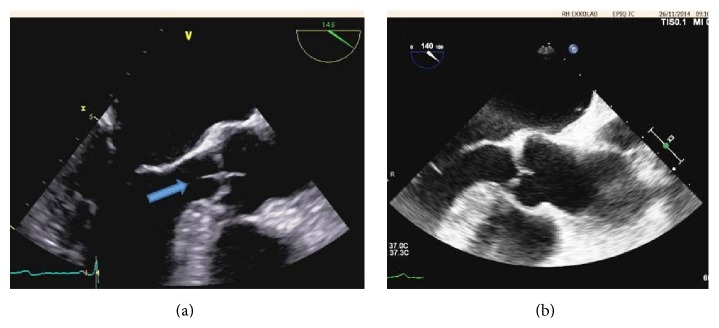
A longitudinal TEE still picture of left ventricle outflow track and the aortic valve. (a) The examination, which led to the surgery, is shown. The arrow marks a thin approximately 10 mm long vegetation with classic location and substance equivalent to Lambl excrescence. (b) A TEE performed after the cancellation and the prior finding is not reproducible.

A 48-year-old man, predisposed to thromboembolic events by smoking, hypertension, hypercholesterolemia, and family history, debuted in 2008 with 5 CT verified cerebral strokes. The patient was treated with platelet inhibitors, antihypertensive, and cholesterol lowering drugs. Due to side effects, the patient stopped all medication after one year. In 2012 and 2014 the patient suffered from strokes again and had a work-up including testing for infectious focus, coronary angiography, and thrombophilia screening. All were negative. Atheromatosis without significant stenosis was found by ultrasound of the carotid arteries. A Transesophageal Echocardiography (TEE) was performed and a thread-like frond was found on the aortic valve (see Figure 1(a)). The excrescence was interpreted as a fibroelastoma and this was thought to explain the strokes. The patient was therefore planned for surgical valve resection. During the anaesthesia a TEE was performed but now the tumour/excrescence was no longer visible. The surgery was therefore aborted. At a TEE performed 3 weeks later it was still not recognized nor was paradox embolism. After reviewing all the TEE data it was concluded that the most likely diagnosis was Lambl excrescence (see Figure 1(b)).

## 3. Discussion

We here present a patient, with multiple predisposing factors for thromboembolic event, who suffered from multiple cerebral infarctions over a 6-year period. Initially a fibroelastoma on the aortic valve was suspected as embolic focus. Due to the classic echocardiographic presentation and to the transient nature Lambl excrescence was assumed to be the most likely diagnosis. LE is common also in healthy individuals and rarely originates a thromboembolic focus. LE should always be considered when assessing excrescences and since LE can be transient, several TEE's tests at different times can be valuable when diagnosing. Finally it is of importance to perform TEE and confirm the diagnosis just before surgical intervention to avoid unnecessary surgery.

## Learning Objective

An excrescence on the aortic valve can be a transient phenomenon. This case shows the need for Transesophageal Echocardiography just before surgical valve replacement.

## References

[1] Roldan C. A., Shively B. K., Crawford M. H. (1997). Valve excrescences: prevalence, evolution and risk for cardioembolism. *Journal of the American College of Cardiology*.

[2] Lambl V. A. (1856). Papillare excrescenzen an der semiluna-klappe er aorta. *Wiener Medizinische Wochenschrift*.

[3] Voros S., Nanda N. C., Thakur A. C., Winokur T. S., Samal A. K. (1999). Lambl's excrescences (valvular strands). *Echocardiography*.

[4] Albuquerque L. C., Trindade V. D. (2011). Heart valve papillary fibroelastoma associated with cardioembolic cerebral events. *Brazilian Journal of Cardiovascular Surgery*.

[5] Sydow K., Willems S., Reichenspurner H., Meinertz T. (2008). Papillary fibroelastomas of the heart. *Thoracic and Cardiovascular Surgeon*.

